# Identification of benign and malignant thyroid nodules based on dynamic AI ultrasound intelligent auxiliary diagnosis system

**DOI:** 10.3389/fendo.2022.1018321

**Published:** 2022-09-27

**Authors:** Bing Wang, Zheng Wan, Chen Li, Mingbo Zhang, YiLei Shi, Xin Miao, Yanbing Jian, Yukun Luo, Jing Yao, Wen Tian

**Affiliations:** ^1^ Senior Department of General Surgery, The First Medical Center of Chinese People's Liberation Army (PLA) General Hospital, Beijing, China; ^2^ Department of Ultrasound, The First Medical Center of Chinese People's Liberation Army (PLA) General Hospital, Beijing, China; ^3^ MedAI Technology (Wuxi) Co. Ltd, Wuxi, China

**Keywords:** thyroid nodules, dynamic artificial intelligence, ultrasonic examination, accurate diagnosis, identification, thyroidectomy

## Abstract

**Background:**

Dynamic artificial intelligence (AI) ultrasound intelligent auxiliary diagnosis system (Dynamic AI) is a joint application of AI technology and medical imaging data, which can perform a real-time synchronous dynamic analysis of nodules. The aim of this study is to investigate the value of dynamic AI in differentiating benign and malignant thyroid nodules and its guiding significance for treatment strategies.

**Methods:**

The data of 607 patients with 1007 thyroid nodules who underwent surgical treatment were reviewed and analyzed, retrospectively. Dynamic AI was used to differentiate benign and malignant nodules. The diagnostic efficacy of dynamic AI was evaluated by comparing the results of dynamic AI examination, preoperative fine needle aspiration cytology (FNAC) and postoperative pathology of nodules with different sizes and properties in patients of different sexes and ages.

**Results:**

The sensitivity, specificity and accuracy of dynamic AI in the diagnosis of thyroid nodules were 92.21%, 83.20% and 89.97%, respectively, which were highly consistent with the postoperative pathological results (kappa = 0.737, *p* < 0.001). There is no statistical difference in accuracy between people with different ages and sexes and nodules of different sizes, which showed the good stability. The accuracy of dynamic AI in malignant nodules (92.21%) was significantly higher than that in benign nodules (83.20%) (*p* < 0.001). The specificity and positive predictive value were significantly higher, and the misdiagnosis rate was significantly lower in dynamic AI than that of preoperative ultrasound ACR TI-RADS (*p* < 0.001). The accuracy of dynamic AI in nodules with diameter ≤ 0.50 cm was significantly higher than that of preoperative ultrasound (*p* = 0.044). Compared with FNAC, the sensitivity (96.58%) and accuracy (94.06%) of dynamic AI were similar.

**Conclusions:**

The dynamic AI examination has high diagnostic value for benign and malignant thyroid nodules, which can effectively assist surgeons in formulating scientific and reasonable individualized diagnosis and treatment strategies for patients.

## Introduction

In the past decade, the incidence rate of thyroid cancer has increased worldwide, and among female patients, the growth rate of thyroid cancer was the fastest in malignant tumor incidence rate (1). At present, the growth trend of thyroid cancer incidence rate in various countries tends to be consistent, and the clinical diagnosis rate of thyroid malignant tumors of various common types and different disease stages is increasing, so the number of thyroid cancer patients admitted to medical institutions has always been rising. Thyroid cancer has become the focus of public safety, but also caused widespread concern in the whole society (2). One of the most important reasons for the rising incidence rate of thyroid cancer worldwide is that, with the popularization of high-resolution ultrasound imaging technology and the enhancement of people’s awareness of physical examination, the detection rate of thyroid nodules has increased significantly, as high as 65%, of which the malignant proportion accounts for about 5%-15% (3).

Ultrasound is the preferred imaging examination to evaluate the benign and malignant thyroid nodules, but the diagnostic results are affected by the factors such as the personal experience of ultrasound doctors, operating skills, ultrasound equipment, and thyroid basic lesions, and the diagnostic accuracy and efficiency of doctors at different levels and with different seniority vary greatly (4–6). It is pointed out, in the guidelines of the American Thyroid Association that combining the characteristics of nodules under ultrasound and using the Bethesda system judgment, the results of fine needle aspiration cytology (FNAC) is the necessary basis for preoperative judgment of benign and malignant thyroid nodules (7), but there are still cases of omission of malignant nodes and unclear pathological diagnosis in the actual clinical diagnosis. A study has shown that about 20% of 25445 biopsy tissue samples were pathologically diagnosed as atypical lesions with unknown significance or could not be determined as malignant, with an average malignant risk of 15.9% and 75.2% respectively (8). With the joint application of artificial intelligence (AI) technology and medical imaging data, static AI ultrasound intelligent auxiliary diagnosis system was generated and used to diagnose thyroid nodules, which can improve the diagnostic efficiency while ensuring the accuracy (9–12). However, static AI can only diagnose a single view of nodules, and cannot judge the nature of nodules in real time. The emergence of dynamic AI ultrasonic intelligent auxiliary diagnosis system (dynamic AI) provides a new method for ultrasonic diagnosis of thyroid nodules, which can carry out real-time synchronous dynamic analysis of nodules from multiple sectional views and different angles, and further improve the efficiency of clinical examination and diagnosis.

In this study, dynamic AI was used to distinguish benign and malignant thyroid nodules in 607 patients with 1007 thyroid nodules, and the diagnostic value and guiding significance of dynamic AI for treatment strategies were explored.

## Patients and methods

### Patients

**Figure 1:**
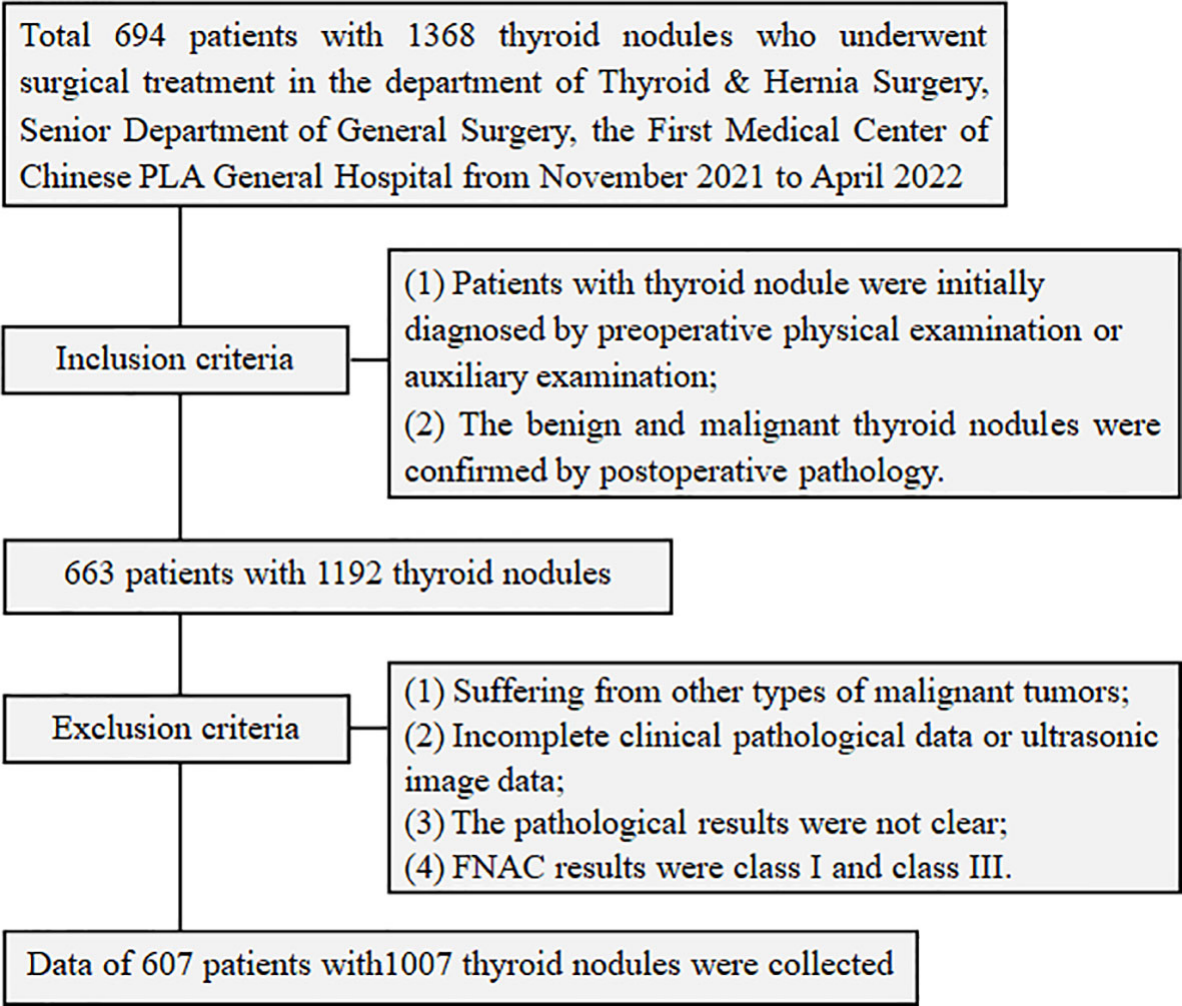
Flowchart of the inclusion and exclusion of patients.

In this study, 607 patients with thyroid nodules who underwent surgical treatment in the department of Thyroid & Hernia Surgery, Senior Department of General Surgery, the First Medical Center of Chinese PLA General Hospital from November 2021 to April 2022 were recruited. Relevant data including the sex, age, pathology of thyroid nodules and results of AI were retrospectively reviewed and collected. All patients met the inclusion criteria, which were as follows (1): Patients with thyroid nodule were initially diagnosed by preoperative physical examination or auxiliary examination; (2) The benign and malignant thyroid nodules were confirmed by postoperative pathology. Exclusion criteria were as follows: (1) Suffering from other types of malignant tumors; (2) Incomplete clinical pathological data or ultrasonic image data; (3) The pathological results were not clear; (4) FNAC results were class I (nondiagnostic or unsatisfactory) and class III (atypia of undetermined significance) (**Figure 1**). This study was approved by the Ethics Committee of Chinese PLA General Hospital (No. S2022-441-01), and written informed consent was obtained from all participants.

### Diagnostic equipment and methods

#### Dynamic AI ultrasonic intelligent examination

Dynamic AI ultrasonic intelligent diagnosis was performed by Ian Thyroid Solution 100 (ITS100) (MedAI Technology Co. Ltd, Wuxi, China). It is an ultrasonic image intelligent system, which is mainly composed of host, GE LOGIQ e ultrasonic diagnostic instrument, GE l4-12t-rs linear array probe and AI auxiliary display screen. All patients were examined by dynamic AI ultrasound intelligent assistant real-time diagnostic system before operation. According to the actual situation of thyroid nodules in different patients, the gain, focus, depth and other parameters of the instrument were properly adjusted to obtain the most satisfactory image data. The patient lied on his back and exposed his neck. The thyroid gland was comprehensively scanned with the probe in the order of left lobe first, then right lobe, from top to bottom, from inside to outside. The transverse and longitudinal sections were scanned for 3 times, respectively, to ensure that no small nodules and small lesions are missed. The examinations of all patients were completed by the same doctor.

#### Routine ultrasound examination

Thyroid ultrasound was performed using an EPIQ 7 (Philips Health Care, Andover, MA, USA) with L125 linear-array transducer (5-12MHz). All patients underwent routine ultrasound examination before operation. The patient lied on his back and exposed his neck. The transverse and longitudinal sections of thyroid gland were scanned with ultrasonic probe in the order of left lobe first, then right lobe, from top to bottom, from inside to outside. The examinations of all patients were completed by the same doctor.

#### FNAC examination

FNAC examination was performed using the Mylab Twice US system (Esaote SpA, Geneva, Italy) with L523 linear-array transducer (4–13 MHz). For patients with suspected malignant nodules by routine ultrasound, further FNAC examination was performed. The patient lied on his back and exposed his neck. The puncture point was selected under ultrasound guidance, a 25G puncture needle was used to enter the center of the nodule under ultrasound guidance. The needle tip was lifted and inserted back and forth for 5-10 times in different parts of the nodule. Then, the needle was quickly withdrawn, and the tissue was taken out to cytological smear for detection. The examinations of all patients were completed by the same doctor.

### Diagnostic criteria

#### Dynamic AI diagnosis

ITS100 can automatically identify the lesion and locate the nodule in real time. Dynamic AI uses computer vision and deep learning technology to establish an artificial intelligence aided diagnosis model for benign and malignant thyroid nodules based on convolutional neural network (CNN). Before the ultrasonic image data is input into the diagnostic model, the preprocessing module processes the image to obtain the image that can be directly used by the model. In the diagnosis model, image features are automatically extracted by using deep learning technology. According to the uniqueness of ultrasonic image features, dynamic AI designs a new CNN structure for the diagnostic model, samples the pixels on the input image with the help of CNN convolution check, obtains the global image features of the thyroid nodule region in the ultrasonic image, constructs a high-throughput, multi-level feature space, and improves the diagnostic performance of the model. After the diagnostic model extracts and calculates the features of the input nodule image, the model outputs two probability values, which respectively represent the probability value that the diagnostic model considers the input nodule to be malignant and benign. When the predicted malignant probability value is greater than or equal to the benign probability value, the predicted result of the model is malignant, showing a red “m” sign. Otherwise, it is benign and a green “B” sign will be shown. The percentage represents the probability. The final dynamic AI diagnosis result is obtained through comprehensive analysis based on the three scanning results. The operation is supervised and guided by an application engineer from AI Company.

#### Routine ultrasound diagnosis

Thyroid nodules were graded according to the thyroid imaging reporting and data system (TI-RADS) issued by the American College of Radiology (ACR) (13), ACR TI-RADS grades 1 ~ 3 were classified as benign and 4 ~ 5 were classified as malignant. After diagnosis by specialized medical personnel in our working group, senior ultrasound doctors in our hospital were invited to review the films.

#### FNAC diagnosis

Thyroid nodule was classified using Bethesda reporting system (14). Class I, unsatisfactory sampling or failure to diagnose; Class II, benign; Class III, atypia of undetermined significance; Class IV, follicular tumor or suspicious follicular tumor; Class V, suspicious malignant tumor; Class VI, malignant tumor. Since FNAC cannot accurately diagnose the benign and malignant of follicular tumors, the follicular tumors can be accurately judged only relying on conventional paraffin pathology to judge whether there is capsule or vascular invasion. Thus, through FNAC, the nodules of class IV can only be judged as benign before operation. Therefore, in this study, class II and IV are judged as benign, and class V and VI were judged as malignant. After diagnosis by specialized medical personnel in our working group, senior pathologists of our hospital were invited to review the films.

### Statistical analysis

The diagnostic efficacy of dynamic AI based on postoperative pathology was calculated, including sensitivity, specificity, accuracy, positive predictive value, negative predictive value, missed diagnosis rate and misdiagnosis rate. Kappa test was used to analyze the consistency of dynamic AI, ACR TI-RADS, FNAC and pathological examination. Kappa value < 0 indicates no significance; Kappa value ≤ 0.40 indicates poor consistency; 0.40 < kappa value ≤ 0.60 indicates moderate consistency; 0.60 < kappa value ≤ 0.80 indicates high consistency; Kappa value > 0.8 indicates very high consistency. A χ^2^ test was used to compare the differences in frequencies between groups, and the data were expressed in percentage. Data of continuous variables are presented as means ± SD. SPSS version 23.0 software (IBM, Chicago, IL, USA) was used for all statistical analyses, and a *P* < 0.05 was considered significant for the difference between groups.

## Results

A total of 607 patients were included in the study, including 160 males and 447 females, aged from 16 to 75 years, with an average age of (43.58 ± 11.47) years, 491 patients < 55 years and 116 patients ≥ 55 years. There were total 1007 thyroid nodules, with a diameter of 0.2 ~ 6.5cm and an average diameter of (1.00 ± 0.91) cm.

### Stability analysis of dynamic AI examination

#### Postoperative pathological confirmation of dynamic AI diagnosis

**Figure 2:**
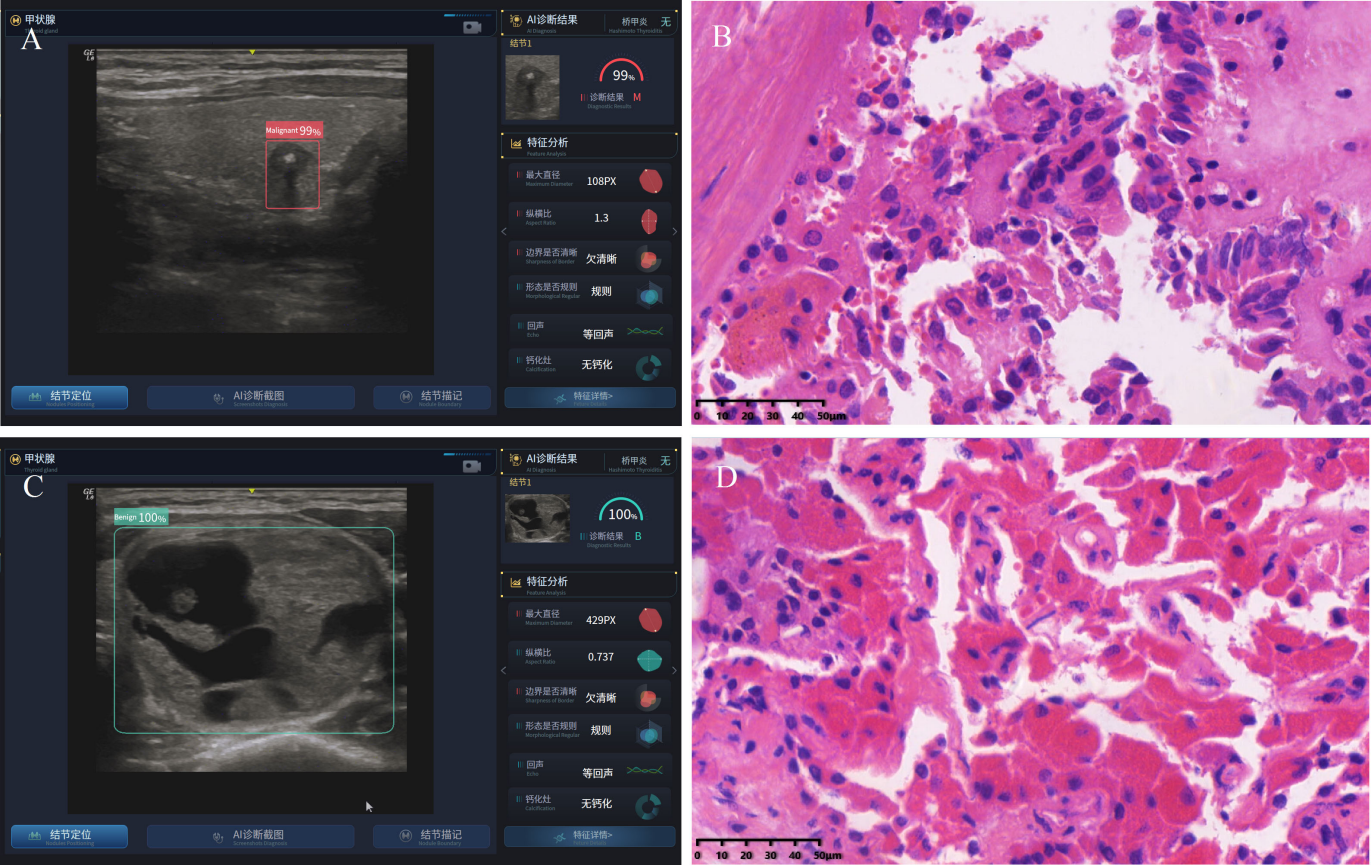
Typical dynamic AI diagnostic plot and postoperative pathology. **(A)** dynamic AI diagnostic plot of thyroid malignant nodule; **(B)** the postoperative pathology of malignant nodule indicates thyroid papillary carcinoma; **(C)** dynamic AI diagnostic plot of benign thyroid nodule; **(D)** the postoperative pathology of benign nodule indicates follicular adenoma.

All nodules (benign and malignant) were confirmed by pathological results after operation (**Figure 2**).

#### Accuracy analysis of dynamic AI examination in different gender groups

Among the 607 patients in this study, 160 were male, 238 of 264 nodules were consistent with the postoperative pathological results, and the accuracy was 90.15%; In 447 women, 668 of 743 nodules were consistent with the postoperative pathological results, with an accuracy of 89.91%; There was no statistical difference in the accuracy of dynamic AI between two gender groups (*p* = 0.909) (**Table 1**).

**Table 1:** Stability analysis of dynamic AI examination.

	Accuracy% (n_1_/n_2_)	*P* values
Gender		0.909
Male	90.15 (238/264)	
Female	89.91 (668/743)	
Age		0.080
< 55 years	91.84 (720/784)	
≥ 55 years	83.41 (186/223)	
Tumor		< 0.001
Malignant	92.21 (698/757)	
Benign	83.20 (208/250)	
Tumor size (maximum diameter, cm)		0.850
Diameter ≤ 0.50	89.18 (305/342)	
0.5 < diameter ≤ 1.0cm	90.32 (364/403)	
1.0 < diameter ≤ 2.0cm	91.81 (157/171)	
2.0 < diameter ≤ 4.0cm	88.06 (59/67)	
Diameter > 4.0 cm	87.50 (21/24)	

AI, artificial intelligence; n_1_, number of nodules accurately diagnosed by dynamic AI; n_2_, total number of nodules.

#### Accuracy analysis of dynamic AI examination in different age groups

Among 607 patients in this study, 491 cases < 55 years, and 720 of 784 nodules were consistent with the postoperative pathological results, with an accuracy of 91.84%; 116 cases ≥ 55 years, 186 of 223 nodules were consistent with the postoperative pathological results, and the accuracy was 83.41%. There was no statistical difference in the accuracy of dynamic AI among different age groups (*p* = 0.080) (**Table 1**).

#### Accuracy analysis of dynamic AI examination in nodules with different pathology

Dynamic AI correctly identified 698 of 757 malignant nodules, with an accuracy of 92.21%; 208 of the 250 benign nodules were accurately identified, with an accuracy of 83.20%, and the difference was statistically significant (*p* < 0.001) (**Table 1**).

#### Accuracy analysis of dynamic AI examination in nodules with different sizes

Among 1007 nodules in this study, the accuracy of dynamic AI in identifying nodules ≤ 0.5cm was 89.18%. The accuracy of identifying nodules 0.5 < diameter ≤ 1.0cm nodules was 90.32%. The accuracy of identifying 1.0 < diameter ≤ 2.0cm nodules was 91.81%. The accuracy of identifying 2.0 < diameter ≤ 4.0cm nodules was 88.06%, and the accuracy of identifying diameter > 4.0cm nodules was 87.50%. The difference was not statistically significant (*p* = 0.850) (**Table 1**).

#### Comparison of diagnostic efficacy between dynamic AI examination and ACR TI-RADS

Among the 1007 nodules in this study, 740 malignant nodules and 267 benign nodules were identified by dynamic AI, of which 698 malignant nodules and 208 benign nodules were consistent with the postoperative pathological results, which were highly consistent with the postoperative pathological results (kappa = 0.737, *p* < 0.001). Preoperative conventional ultrasound ACR TI-RADS grading showed 793 partial malignant nodules and 214 partial benign nodules, of which 712 malignant nodules and 169 benign nodules were consistent with postoperative pathological results (kappa = 0.648, *p* = 0.029). These results showed that the consistency between dynamic AI and postoperative pathology was higher than that of preoperative conventional ultrasound (**Table 2**). The comparative analysis of the diagnostic efficacy between dynamic AI and ACR TI-RADS showed that the specificity and positive predictive value of dynamic AI were significantly higher, and the misdiagnosis rate was significantly lower than that of preoperative ultrasound ACR TI-RADS (*P* < 0.001) (**Table 3**).

**Table 2:** Consistency analysis of dynamic AI, ACR TI-RADS, FNAC and pathological examination.

	Postoperative pathology (n)		*Kappa*	*P* values
	Malignant	Benign		
Total patient dynamic AI			0.737	< 0.001
Malignant	698	42		
Benign	59	208		
ACR TI-RADS			0.648	0.029
Malignant	712	81		
Benign	45	169		
FNAC				
Malignant	614	4	0.686	< 0.001
Benign	30	42		

AI, artificial intelligence; ACR TI-RADS, thyroid imaging reporting and data system issued by the American College of Radiology; FNAC, fine needle aspiration cytology.

**Table 3:** Comparison of diagnosis efficiency between dynamic AI and ACR TI-RADS.

	Dynamic AI (%)	ACR TI-RADS (%)	*P* values
Sensitivity	92.21 (698/757)	94.06 (712/757)	0.155
Specificity	83.20 (208/250)	67.60 (169/250)	< 0.001
Accuracy	89.97 (906/1007)	87.49 (881/1007)	0.078
Positive predictive value	94.32 (698/740)	89.79 (712/793)	0.001
Negative predictive value	77.90 (208/267)	78.97 (169/214)	0.777
Missed diagnosis rate	7.79 (59/757)	5.94 (45/757)	0.155
Misdiagnosis rate	16.80 (42/250)	32.40 (81/250)	< 0.001

AI, artificial intelligence; ACR TI-RADS, thyroid imaging reporting and data system issued by the American College of Radiology; Sensitivity, number of accurately diagnosed malignant nodules/total number of malignant nodules; Specificity, number of accurately diagnosed benign nodules/total number of benign nodules; Accuracy, number of accurately diagnosis nodules/total number of nodules; Positive predictive value, number of accurately diagnosed malignant nodules/number of diagnosed malignant nodules; Negative predictive value, number of accurately diagnosed benign nodules/number of diagnosed benign nodules; Missed diagnosis rate, number of misdiagnosed malignant nodules/total number of malignant nodules; Misdiagnosis rate, number of misdiagnosed benign nodules/total number of benign nodules.

#### Comparison of diagnostic efficacy between dynamic AI examination and FNAC

Among the 1007 nodules in this study, 690 were diagnosed by dynamic AI and FNAC before operation. Dynamic AI examination identified 641 malignant nodules and 49 benign nodules, of which 622 malignant nodules and 27 benign nodules were consistent with postoperative pathological results. FNAC diagnosed 618 malignant nodules and 72 benign nodules, of which 614 malignant nodules and 42 benign nodules were consistent with the postoperative pathological results (kappa = 0.686, *P* < 0.001) (**Table 2**). The comparative analysis of the diagnostic efficacy between dynamic AI and FNAC showed that the sensitivity and accuracy of dynamic AI were equivalent to that of FNAC, but the FNAC showed the higher positive predictive value and lower misdiagnosis rate compared with dynamic AI (*P* < 0.05) **(** **Table 4** **)**.

**Table 4:** Comparison of diagnostic efficacy between dynamic AI examination and FNAC.

	Dynamic AI (%)	FNAC (%)	*P* values
Sensitivity	96.58 (622/644)	95.34 (614/644)	0.257
Specificity	58.70 (27/46)	91.30 (4/46)	< 0.001
Accuracy	94.06 (649/690)	95.07 (656/690)	0.406
Positive predictive value	97.04 (622/641)	99.35 (614/618)	0.002
Negative predictive value	55.10 (27/49)	58.33 (42/72)	0.724
Missed diagnosis rate	3.40 (22/644)	4.66 (30/644)	0.257
Misdiagnosis rate	41.30 (19/46)	8.70 (4/46)	< 0.001

AI, artificial intelligence; FNAC, fine needle aspiration cytology; Sensitivity, number of accurately diagnosed malignant nodules/total number of malignant nodules; Specificity, number of accurately diagnosed benign nodules/total number of benign nodules; Accuracy, number of accurately diagnosis nodules/total number of nodules; Positive predictive value, number of accurately diagnosed malignant nodules/number of diagnosed malignant nodules; Negative predictive value, number of accurately diagnosed benign nodules/number of diagnosed benign nodules; Missed diagnosis rate, number of misdiagnosed malignant nodules/total number of malignant nodules; Misdiagnosis rate, number of misdiagnosed benign nodules/total number of benign nodules.

#### Accuracy analysis of dynamic AI, ACR TI-RADS and FNAC in diagnosis of nodules with different diameters

In this study, the accuracy of dynamic AI in nodules ≤ 0.5cm was significantly higher than that of preoperative conventional ultrasound (*P* < 0.05). For the nodule in diameter ≤ 0.50 cm, 0.5 < diameter ≤ 1.0cm and 2.0 < diameter ≤ 4.0cm, the accuracy rate was higher than that of preoperative conventional ultrasound, but with no statistical significance (**Table 5**). Among 690 nodules diagnosed by preoperative dynamic AI examination, ACR TI-RADS and FNAC, there was no statistical difference in the diagnostic accuracy among the three groups (**Table 6**).

**Table 5:** Accuracy analysis of dynamic AI and ACR TI-RADS in nodules with different diameters.

Nodule diameter (cm)	Dynamic AI% (n_1_/n_2_)	ACR TI-RADS% (n_3_/n_2_)	*P* values
Diameter ≤ 0.50	89.18 (305/342)	83.92 (287/342)	0.044
0.5 < diameter ≤ 1.0	90.32 (364/403)	89.08 (359/403)	0.562
1.0 < diameter ≤ 2.0	91.81 (157/171)	92.98 (159/171)	0.683
2.0 < diameter ≤ 4.0	88.06 (59/67)	80.60 (54/67)	0.235
Diameter > 4.0	87.50 (21/24)	91.67 (22/24)	0.637

AI, artificial intelligence; ACR TI-RADS, thyroid imaging reporting and data system issued by the American College of Radiology. n_1_, number of nodules accurately diagnosed by dynamic AI; n_2_, total number of nodules; n_3_, number of nodules accurately diagnosed by routine ultrasound before operation.

**Table 6:** Accuracy analysis of dynamic AI, ACR TI-RADS and FNAC diagnosis in 690 nodules with different diameters.

Nodule diameter (cm)	Accuracy			*P* values
	Dynamic AI% (n_1_/n_2_)	ACR TI-RADS% (n_3_/n_2_)	FNAC% (n_4_/n_2_)	
Diameter ≤ 0.5	93.64 (206/220)	95.91 (211/220)	94.09 (207/220)	0.540
0.5 < diameter ≤ 1.0	94.74 (288/304)	94.08 (286/304)	96.05 (292/304)	0.527
1.0 < diameter ≤ 2.0	95.97 (119/124)	96.77 (120/124)	95.16 (118/124)	0.812
Diameter > 2.0	85.71 (36/42)	85.71 (36/42)	92.86 (39/42)	0.506

AI, artificial intelligence; ACR TI-RADS, thyroid imaging reporting and data system issued by the American College of Radiology; FNAC, fine needle aspiration cytology. n_1_, number of nodules accurately diagnosed by dynamic AI; n_2_, total number of nodules; n_3_, number of nodules accurately diagnosed by routine ultrasound before operation; n4, number of nodules accurately diagnosed by FNAC before operation.

## Discussion

AI is a new discipline that simulates human consciousness and thinking process. At present, it has been widely used in the medical field. It plays a vital role in the drug design and discovery (15, 16), diagnosis, treatment, outcome prediction and prognosis evaluation of diseases, the construction of hospital informatization (17–20), as well as diagnosis and management of various endocrine diseases (21). It has changed the medical model and promoted the development of medicine. AI assisted imaging diagnosis is the fastest growing and most widely used field in clinical practice. AI assisted ultrasound diagnosis can realize intelligent ultrasound prenatal examination (22), intelligent cardiac ultrasound examination (23), intelligent diagnosis of thyroid nodules (9–12) and breast nodules (24), etc. It has become increasingly mature and has the uniqueness of non-invasive, convenient and reproducible.

Thyroid gland has become the precursor of AI development in the field of ultrasound due to its unique superficial position and relatively easy to collect standard images (25). AI ultrasound intelligent auxiliary diagnosis system based on static ultrasound images can realize automatic delineation of nodules, morphological recognition, benign and malignant discrimination, and has high diagnostic value for the judgment of benign and malignant thyroid nodules (9–12). On this basis, dynamic AI uses super large-scale convolution neural network (CNN) and deep learning technology to establish an artificial intelligence aided diagnosis model for benign and malignant thyroid nodules based on CNN to automatically extract image features. According to the uniqueness of ultrasonic image features, it designs a new convolution neural network structure for the diagnosis model, obtains the global image features of thyroid nodules in ultrasonic images, and constructs a high-throughput and multi-level feature space to improve the diagnostic performance of the model, and realize the real-time localization, real-time sketch and real-time diagnosis of thyroid nodules in the examination process.

In the study, dynamic AI was used to judge the benign and malignant of 1007 thyroid nodules. There was no statistical difference in the accuracy of different age and sex groups and in different size nodules, which indicated that the stability of dynamic AI was good. The accuracy of dynamic AI was 92.21% in malignant nodules and 83.20% in benign nodules. The difference was statistically significant, which indicated that dynamic AI examination was more accurate in the diagnosis of malignant nodules and slightly inferior in the diagnosis of benign nodules. There are two reasons. First, patients with benign nodules often adopted the treatment mode of reexamination and follow-up. Only a few patients who met the surgical indications underwent surgical resection and pathological diagnosis, Therefore, dynamic AI can collect less learning data for benign nodules. In future work, it is necessary to strengthen the in-depth learning and training for benign nodules, improve the accuracy of benign nodule diagnosis, and promote the improvement of dynamic AI ultrasonic intelligent auxiliary diagnosis system. Second, it may be limited by the number of samples, and large sample size research that will be conducted in the future.

The sensitivity, specificity and accuracy of dynamic AI were 92.21%, 83.20% and 89.97% respectively, which were highly consistent with postoperative pathological results (*kappa* = 0.737, *p* < 0.001), and had high diagnostic value for benign and malignant nodules. The consistency between dynamic AI and postoperative pathology was higher than that of preoperative conventional ultrasound ACR TI-RADS grading (*kappa* = 0.648, *p* = 0.029). A study showed that the accuracy rate of thyroid nodules diagnosed by doctors in hospitals through ultrasound images is about 70% (26). The present study showed that the specificity and positive predictive value of dynamic AI were significantly higher, and the misdiagnosis rate was significantly lower than that of preoperative ultrasound ACR TI-RADS, which suggested that the dynamic AI has a stronger diagnostic ability for benign nodules, thus reducing the misdiagnosis rate, reducing unnecessary puncture biopsy, and saving medical resources and social costs. In this study, the accuracy of dynamic AI in nodules with diameter ≤ 0.50 cm, 0.5 < diameter ≤ 1.0cm and 2.0 < diameter ≤ 4.0cm was higher than that of preoperative conventional ultrasound. The accuracy rates of groups were stable at 87.50% ~ 91.81%, with small fluctuations, while the accuracy of preoperative conventional ultrasound fluctuated between 80.60%~92.98%, and the accuracy in nodules with diameter ≤ 0.5cm was only 83.92%. The difference was statistically significant, which indicated that dynamic AI has high diagnostic value in judging the benign and malignant of micro nodules. This part of nodule is easy to be missed and misdiagnosed in clinical practice, which is the focus and difficulty of clinical diagnosis. In addition, the dynamic AI examination is easy to operate and the physician’s learning curve is short. Before the operation, the surgeon can observe the relationships between the nodules and the capsule, trachea, esophagus, surrounding muscle and adipose tissue through the dynamic AI examination, assist in assessing the risk of contralateral nodules with uncertain nature, and make a good condition assessment. Therefore, a scientific and reasonable individualized diagnosis and treatment strategy before the operation can be formulated, which is also more conducive to better communicate with the patient and his family members and reduce medical disputes. During the operation, the resection scope was determined according to the tumor conditions and the dynamic AI results before the operation.

Among the 690 nodules with needle aspiration before operation, the sensitivity (96.58%) and accuracy (94.06%) of dynamic AI were equivalent to that of FNAC. But, there were statistical differences in specificity, positive predictive value and misdiagnosis rate, which indicated that dynamic AI was inferior to FNAC in the diagnosis of benign nodules. This reason has been mentioned above, mainly because dynamic AI collected less learning data for benign nodules than malignant nodules. With the accumulation of the number of patients and the strengthening of the follow-up in-depth learning and training of benign nodules, the dynamic AI ultrasound intelligent auxiliary diagnosis system will be more perfect, and the accuracy of benign nodule diagnosis will be further improved. In the study on the diagnostic accuracy of dynamic AI, preoperative ultrasound and FNAC in nodules with different diameters, there was no statistical difference among the three groups, and the diagnostic efficiency was equivalent, especially for nodules with diameter ≤ 0.50 cm, the diagnostic accuracy reached 93.64%. However, it was difficult to finish the needle aspiration on these nodules in clinical practice. It can be seen that dynamic AI has high diagnostic value in judging the nature of small nodules. In this part, we did not group the > 4cm nodules separately, because there were only 4 nodules with diameter > 4.0cm in the preoperative pathology through needle aspiration, and the sample size was small. In the future, we will increase the sample size to further study this part. Although FNAC diagnosis can be used as the gold standard for preoperative diagnosis, there are still cases of missed malignant nodules and unclear pathological diagnosis in the actual clinical diagnosis. In addition, the operation of preoperative needle aspiration examination is complex. It is difficult to carry out in areas with relatively backward medical technology resources or grass-roots hospitals. Improper operation may cause complications such as bleeding, nerve injury, and tracheoesophageal injury. The diagnostic accuracy of dynamic AI examination is equivalent to that of FNAC, it also has the characteristics and advantages of safety, non-invasive, high efficiency and convenience, good repeatability, simple operation, and more saving medical resources and social costs than puncture examination.

## Conclusion

This study shows that dynamic AI examination has high diagnostic value for benign and malignant thyroid nodules. The AI system based on big data has the advantages of objectivity, safety, noninvasive, efficiency, convenience and accuracy. Non ultrasound professionals can also easily grasp the operation methods and have a short learning curve. It can assist surgeons in formulating scientific and reasonable individualized diagnosis and treatment strategies and surgical resection scope for patients, and reduce unnecessary puncture biopsy and possible complications, save medical resources and social costs, and reduce medical expenses. It is worth popularizing in clinic. It should be noted that AI can only be used as a clinical auxiliary diagnostic tool at this stage due to the safety, medical ethics, responsibility division, and privacy protection (27). With the continuous exploration of medical data, we believe that AI will show a broader application prospect in the medical field, which is expected to assist in the intelligent and accurate diagnosis of benign and malignant thyroid nodules and effectively assist in guiding clinical diagnosis and treatment strategies.

## Data availability statement

The raw data supporting the conclusions of this article will be made available by the authors, without undue reservation.

## Ethics statement

The studies involving human participants were reviewed and approved by Ethics Committee of Chinese PLA General Hospital. The patients/participants provided their written informed consent to participate in this study.

## Author contributions

JY and WT conceptualized and designed the study. BW, ZW, CL, MZ, YS, and YL collected the data. XM and YJ analyzed the data. BW wrote and edited the paper. All authors contributed to the article and approved the submitted version.

## Conflict of interest

Author YS is employed by MedAI Technology (Wuxi) Co. Ltd.

The remaining authors declare that the research was conducted in the absence of any commercial or financial relationships that could be construed as a potential conflict of interest.

## Publisher’s note

All claims expressed in this article are solely those of the authors and do not necessarily represent those of their affiliated organizations, or those of the publisher, the editors and the reviewers. Any product that may be evaluated in this article, or claim that may be made by its manufacturer, is not guaranteed or endorsed by the publisher.
